# Daily feed intake patterns of purebred nucleus boars as genetic indicators for disease resilience of crossbred barrows under a natural polymicrobial disease challenge

**DOI:** 10.1093/jas/skaf357

**Published:** 2025-10-15

**Authors:** Mostafa Madad, John C S Harding, Michael K Dyck, Frederic Fortin, Graham S Plastow, PigGen Canada, Tom Rathje, Bob Kemp, Daniela Grossi, Egbert Knol, Patrick Charagu, Jack C M Dekkers

**Affiliations:** Department of Animal Science, Iowa State University, Ames, IA 50011; Department of Large Animal Clinical Science, University of Saskatchewan, Saskatoon, SK S7N 5A2, Canada; Department of Agriculture, Food and Nutritional Science, University of Alberta, Edmonton, AB T6G 2R3, Canada; Centre de Développement du Porc du Québec Inc, Québec City, G1V 4M6, Canada; Department of Agriculture, Food and Nutritional Science, University of Alberta, Edmonton, AB T6G 2R3, Canada; PigGen Canada Research Consortium, Guelph, Ontario N1H4G8, Canada; Department of Agriculture, Food and Nutritional Science, University of Alberta, Edmonton, AB T6G 2R3, Canada; Department of Animal Science, Iowa State University, Ames, IA 50011

**Keywords:** daily feed intake, disease resilience, genetic correlation, heritability, pigs

## Abstract

Resilience is an important selection target in pig production to reduce the impact of stressors on performance and welfare, in particular disease stressors. Disease resilience is, however, difficult to select for because purebred selection candidates must be raised under high biosecurity. Previous research showed that patterns of feed intake and feeding behavior of pigs under a disease challenge are genetically correlated with disease resilience. Given the wealth of individual feed intake data that is collected on purebred selection candidates, the objective of this study was to determine whether patterns of feed intake derived from such data can be used as genetic indicators to select for disease resilience of crossbred pigs. Daily feed intake on 27,880 boars from 5 Landrace and Large White breeding populations were used to derive three potential disease resilience indicators: the square root of the standard deviation (RSD), the lag-one autocorrelation (AC), and the skewness (SK) or residuals of linear regression of feed intake on age. Heritability estimates were 0.13 for RSD, 0.08 for AC, and 0.06 for SK. Estimates of genetic correlations with growth rate and feed intake of these same purebreds were high positive for RSD, close to zero for AC, and moderate negative for SK. Estimates of genetic correlations of the purebred traits with traits of their crossbred barrows (*n* = 1,818) that were exposed to a natural polymicrobial disease challenge indicated that resilience measures derived from purebred nucleus data are different genetic traits than similar measures (i.e., RSD) on their crossbreds under disease, as are corresponding performance traits such as growth and feed intake. Estimates of genetic correlations of the three indicator traits of purebreds with resilience traits of crossbreds under the disease challenge, including growth rate, mortality, and veterinary treatment rates, were highly variable and on average close to zero. We conclude that the purebred feed intake pattern traits evaluated here are not ready to be used to select for disease resilience because of inconsistent results and large standard errors of genetic correlation estimates. However, results do suggest that resilience measures derived from feed intake and behavior traits (e.g., based on duration) of purebreds in high-health nucleus herds may contain information that is genetically correlated to disease resilience in the field. Additional research is needed to identify such measures.

## Teaser

Daily feed intake and feeding behavior data collected on purebred pigs in high-health nucleus herds may contain information that can be used to select for improved disease resilience of their crossbred offspring in the field, without having to expose purebred selection candidates to disease. However, the measures and investigated here and alternate measures that can be derived from such data require further research to confirm their genetic correlations with disease resilience in the field before they can be recommended for use in selection programs.

## Introduction

During their lifetime, pigs face various infectious challenges, often simultaneously, and concurrent with non-infectious challenges, and how they are able to cope with these challenges is an important determinant of their productivity and care requirements and also has important animal welfare implications. Strategies to reduce the incidence and impact of infectious disease include biosecurity, vaccination, therapeutic and metaphylactic medications, as well as genetic improvement. With regard to the latter, several have argued that genetic selection should focus on disease resilience, rather than on resistance or tolerance to infectious pathogens (Mulder and Rashidi, 2017; Knap and Doeschl-Wilson, 2020). In general, resilience is defined as the capacity of an animal to be minimally affected by disturbances or to rapidly return to the state pertained before exposure to a disturbance (Colditz and Hine, 2016, Homma et al., 2021). Disease resilience refers the ability of an animal to maintain performance in the face of pathogen exposure and to require fewer veterinary treatments and attention time (Berghof et al., 2019b).

Incorporating disease resilience in swine breeding programs is challenging because nucleus breeding stock are reared under high biosecurity. Although strategies have been proposed for the collection of disease-related phenotypes on commercial farms and for their use for selection at the nucleus level, these are difficult and costly to implement (Knap, 2005), in part because it requires phenotypes on individual animals that either have pedigree recorded back to the nucleus or that are genotyped. For a recent review of such strategies, see Dekkers (2025). Nevertheless, if such data can be obtained, several studies have shown that disease-related phenotypes that have moderate heritabilities can be identified (Henryon et al., 2001, Gorssen et al., 2021), although some studies have found these traits to have low heritability (Hermesch et al., 2017; Guy et al., 2018; Lenoir et al., 2022), unless disease incidence is high, as in, for example, Putz et al. (2019).

Several recent studies have proposed that patterns and fluctuations of an animal’s phenotypes over time for longitudinal traits, such as milk yield, egg production, body weight, and feed intake, can be used to derive indicators of resilience (Mulder, 2017; Scheffer et al., 2018; Berghof et al., 2019b). These are typically derived based on residuals from functions of time or age that are fitted to an individual’s longitudinal data and include day-to-day variation (animals with larger variation are less resilient), skewness (animals with more negative residuals for, e.g., feed intake, are less resilient), the auto-correlation of residuals over time (animals with a negative autocorrelation of residuals are less resilient) (Berghof et al., 2019b), or the proportion of residuals that are considered negative outliers (e.g., off-feed days, as proposed by Putz et al. (2019)).

Using feed intake data on finisher pigs exposed to a severe polymicrobial natural disease challenge, Putz et al. (2019) and Cheng et al. (2020, 2021) showed that day-to-day variation in feed intake or duration and the proportion of off-feed days based on feed intake or duration were low to moderately heritable (0.08 to 0.23) and genetically positively correlated with veterinary treatment rates and mortality under a natural polymicrobial disease challenge (up to 0.59 ± 0.32 and 0.69 ± 0.26, respectively) and that they tended to be negatively correlated with growth rate under the disease challenge (up to −0.39 ± 0.26). Cheng et al. (2021) found that similar traits derived from drinking data from these same pigs did not have consistent genetic correlations with disease resilience traits but that water intake duration and the number of visits to the drinker did have negative genetic correlation estimates with treatment and mortality rates (up to −0.45 ± 0.19). Gorssen et al. (2024) evaluated residuals for biweekly body weights of commercial crossbred pigs in an experimental farm with commercial conditions, including presence of disease, and found their variation to be moderately heritable (0.25 to 0.36). They also estimated deviations in body weight to have positive genetic correlations with tail biting wounds (0.22 to 0.30), lameness (0.15 to 0.31), and mortality (0.19 to 0.33).

In the above studies, these indicators traits of resilience were derived from feed intake data collected under conditions that mimicked those present in commercial farms, with high disease pressure in the case of Putz et al. (2019) and Cheng et al. (2020, 2021), which are typically not available. However, extensive individual feed intake is routinely collected on purebred selection candidates in nucleus herds, that is, under high-health conditions. Thus, derivation of resilience indicator traits from these data would allow the direct estimation of breeding values on the purebred selection candidates. To investigate the heritability of such potential disease resilience traits measured on purebreds in nucleus environments, Li et al. (2024) investigated the four resilience measures proposed by Putz et al. (2019) in a purebred Duroc population of limited size (*n* = 550) and estimated their heritabilities to range from 0.09 to 0.25. Gorssen et al. (2023) derived resilience traits using longitudinal body weight, feed intake, and feeding behaviour data from a purebred population of Piétrain pigs in a nucleus environment and found them to be lowly to moderately heritable (estimates ranging from 0.03 to 0.28). Mancin et al. (2024) also found that resilience measures derived from feed intake data on Duroc boars in a nucleus environment were lowly to moderately heritable (0.08 to 0.17).

With some exceptions, most studies investigating resilience indicators derived on purebred nucleus data were not able to determine whether the resulting traits were genetically correlated with disease resilience, that is, performance under disease. Harlizius et al. (2020) found that estimated breeding values (EBV) of purebred boars for day-to-day variation of feed intake in the nucleus were positively correlated with mortality of their progeny in an experimental disease challenge. This study was, however, based on limited numbers of animals. Kavlak and Uimari (2024) investigated the resilience indicator traits proposed by Putz et al. (2019), along with the coefficient of variation for daily feed intake and duration using data from Finnish Yorkshire and Landrace pigs and their F1 crossbred pigs raised in a central test station. They found that EBV for day-to-day variation in feeding time had a positive association with the number of sick days in the central test station, although the incidence of pigs identified as ‘sick’ was low and almost half of the sick pigs were identified based on limp, tail biting, or skin damage. Finally, Mancin et al. (2024) found that genomic regions associated with some resilience measures derived from feed intake of Duroc boars in a nucleus environment colocalized with genes associated with immune response and PRRSV response. Although these studies are promising, addition evidence is needed before potential disease indicator traits derived from feed intake take data from purebred pigs in nucleus herds can be used to improve disease resilience of crossbred pigs at the commercial level.

Against this background, the objective of this study was to determine whether pattern traits derived from longitudinal feed intake data collected on purebred animals in nucleus herds can be used as indicator traits to select for disease resilience of crossbred pigs. To this end, we used data from the polymicrobial natural disease challenge model (NDCM) for crossbred Large White x Yorkshire barrows described by Putz et al. (2019) and Cheng et al. (2020), as well as nucleus data on the purebred parental lines of some of these pigs to estimate genetic parameters of potential resilience indicator traits derived from feed intake data collected in the nucleus and their genetic correlations with performance of their crossbreds under the disease challenge.

## Materials and Methods

### Populations and data collection

The NDCM was carried out following the Canadian Council on Animal Care guidelines (https://www.ccac.ca/en/certification/about-certification/). The protocol was approved by the Protection Committee of the Centre de Recherche en Sciences Animales de Deschambault and the Animal Care and Use Committee at the University of Alberta (AUP00002227). The project was overseen by the herd veterinarian of the Centre de développement du porc du Québec (CDPQ) together with the research project veterinarians. The purebred data analyzed in this study was collected as part of the routine breeding programs of member companies of PigGen Canada.

Data on the purebred parental lines from two of the seven companies that provided Large White x Landrace barrows for the NDCM were used. Data from company 1 was on 2,432 Landrace (LR1) nucleus boars and 4,053 Large White (LW1) nucleus boars born from 2010 to 2020, and on 890 of their crossbred barrows that were entered into the NDCM between 2015 and 2021 in 13 batches of 60 or 75 pigs. For company 2, data were available for two Large White purebred nucleus populations (LW2a and LW2b) and one Landrace nucleus population (LR2), with data on, respectively, 9,149, 4,075, and 8,171 boars, born between 2016 and 2021, along with data on 945 of their crossbred barrows that were entered into the NDCM in 14 batches. The purebred data were collected as part of the routine breeding programs of each company. As described by Putz et al. (2019) and Cheng et al. (2020), in the NDCM, a batch of 60 or 75 weaned piglets from one healthy multiplier from one company, in rotation, was entered into a quarantine nursery (qNur) every 3 weeks. After 3 weeks, they were moved into the challenge nursery (cNur) for 28 d and then to the finisher (Fin) until they reached market weight. The challenge nursery and finisher were set up in a research barn with common air space at CDPQ in Quebec, Canada, by initially introducing sick pigs from neighboring farms, resulting in the presence of multiple bacterial, viral, and parasitic pathogens in the barn. The disease challenge was maintained using continuous flow and horizontal transmission during the 1-week overlap of the new batch with the previously entered batch in the challenge nursery.

### Performance and resilience traits

Phenotypes available on the purebred boars were from the standard growth performance testing program of each company and included daily feed intake data collected using single space electronic feeders, average daily gain (ADG), and ultrasound backfat (FAT) and lean depth (LEAN). For company 1, electronic feeders were switched weekly between two pens. Data from the days of the switch were excluded, as were the initial days of feeder use for both companies, to allow for acclimation. Only records of pigs with at least 20 d of feed intake data were kept in the analysis. We also removed pigs with age at the first available daily feed intake record less than 50 or greater than 100 d and with age at the last available daily feed intake record less than 70 or greater than 200 d. Daily records with feed intake equal to 0 or greater than 5.5 kg were also deleted. The average age at the first and last feed intake record was 88 and 161 d, respectively, for company 1 and 77 and 149 d for company 2.

Several potential resilience indicator traits were derived from residuals of the linear regression of daily feed intake data that were available for each purebred boar tested on age. The utilized metrics encompassed the root mean square error of residuals (SD), the lag-one autocorrelation (AC) of residuals, and the skewness of residuals (SK), as proposed by Berghof et al. (2019b). Descriptive statistics of the resulting phenotypes, along with average daily feed intake (ADFI) are shown in Table 1. Because the distribution of SD was skewed, it was also analyzed after taking its square root, which will be referred to as root SD (RSD). For the crossbred barrows, the performance and resilience phenotypes collected in the NDCM, as described by Putz et al. (2019) and Cheng et al. (2020), were available. Resilience indicators computed for crossbreds included included the proportion of off-feed days based on feed intake (OFF_FI_) or feed intake duration (OFF_DUR_) and day-to-day variation in feed intake (VAR_FI_) or feed intake duration (VAR_DUR_). As described by Putz et al. (2019), off-feed days were identified based on negative residuals for 5% quantile regression of daily feed intake or duration on age across all pigs. Day-to-day variation measures were based on the root mean square error of within-individual regression of daily feed intake or duration on age. Descriptive statistics of these traits are in Table 2.

**Table 1.** Summary statistics for daily feed intake and resilience indicator traits on boars from five purebred nucleus populations

Population	Trait^1^	Number of boars	Mean	Standard deviation
**LR1**	SD	2,478	0.32	0.18
	AC		0.50	0.20
	SK		0.05	0.56
**LR2**	SD	8,491	0.33	0.18
	AC		0.39	0.23
	SK		−0.08	0.40
**LW1**	SD	4,147	0.32	0.20
	AC		0.41	0.23
	SK		0.03	0.64
**LW2a**	SD	9,350	0.35	0.18
	AC		0.49	0.20
	SK		0.03	0.37
**LW2b**	SD	4,152	0.32	0.17
	AC		0.00	0.17
	SK		0.07	0.40

1 Resilience indicator traits derived from residuals of linear regression of longitudinal feed intake on age: SD = standard deviation (kg/day); AC = first order auto-correlation; SK = skewness.

**Table 2.** Summary statistics of daily feed intake and resilience traits for crossbred pigs from Companies 1 and 2 in the natural disease challenge

	Company 1			Company 2		
**Trait** ^1^	Number of animals	Mean	Standard deviation	Number of animals	Mean	Standard deviation
**qNurADG, kg/d**	885	0.35	0.11	937	0.27	0.09
**cNurADG, kg/d**	881	0.31	0.19	933	0.28	0.14
**FinADG, kg/d**	629	0.91	0.16	798	0.87	0.13
**AllMOR**	890	0.28	0.45	945	0.17	0.38
**NurMOR**	885	0.11	0.32	938	0.07	0.25
**nTrtsNur2_27**	870	1.14	1.38	927	0.67	0.91
**nTrtsFin_100**	608	0.30	0.55	781	0.20	0.47
**AllTRT**	610	1.17	1.24	781	0.81	1.00
**ADFI, kg/d**	608	2.38	0.35	773	2.16	0.31
** VAR_FI_, kg**	608	0.50	0.11	780	0.47	0.10
**VAR_DUR_ ,min**	608	12.50	3.46	780	12.50	3.50
**OFF_FI_**	603	0.03	0.05	777	0.04	0.06
**OFF_DUR_**	603	0.03	0.04	777	0.04	0.05

1 qNurADG, average daily gain in quarantine nursery, cNurADG, average daily gain in challenge nursery, FinADG, average daily gain in finisher, AllMOR, mortality rate for pigs in challenge nursery and finisher, NurMOR, mortality rate for pigs in challenge nursery, nTrtsNur2_27, nTrtsFin_100, AllTRT, number of health treatments per pig in challenge nursery and finisher, ADFI, feed intake, VAR_FI,_ day-to-day variation in feed intake, VAR_DUR_, day-to-day variation in duration, OFF_FI_, proportion of off-feed days based on 5% quantile regression for feed intake, OFF_DUR_, proportion of off-feed days based on 5% quantile regression for feed intake duration.

All purebred pigs were genotyped using custom 50k Affymetrix Axiom Porcine Genotyping Arrays by each company. Crossbred animals were genotyped using a 650k Affymetrix Axiom Porcine Genotyping Array, as described by Putz et al. (2019). The software FImpute (Sargolzaei et al., 2014) was used to impute the genomic marker set for purebred pigs from 50k to 650k SNPs, aligning it with the size of the marker set available for crossbred pigs.

### Statistical analyses

Single-trait mixed linear animal models with genomic relationships were used to estimate heritabilities of all traits, separately for each population, and corresponding two-trait mixed linear animal models were used to estimate genetic correlations within and between populations, using ASReml 4.2 (Gilmour et al., 2022). The model used for genetic analyses of the performance and resilience indicator traits of each purebred population was:


$$y_{\mathrm{iklmn}}={CG}_{i}+{\mathrm{Litter}}_{l}+u_{m}+e_{\mathrm{iklmn}}$$

where $y_{\mathrm{ijklmn}}$ is a phenotype, ${CG}_{i}$ is the fixed effect of contemporary group, which included batch and feed intake station for the LR1 and LW1 populations, and herd, year, week, and feeder for the LR2, LW2a and LW2b populations, ${\mathrm{Litter}}_{l}$ is the random environmental effect of litter, $u_{m}$ is the random additive genetic effect, and $e_{\mathrm{ijklmn}}$ is the residual effect. The mixed linear animal model used to analyze the traits for each crossbred population was as described by Cheng et al. (2020):


$$y_{\mathrm{iklmn}}={\mathrm{Batch}}_{i}+{\mathrm{Age}}_{k}+{\mathrm{Pen}}_{l}+{\mathrm{Litter}}_{m}+u_{n}+e_{\mathrm{iklmn}}$$

where $y_{\mathrm{ijklmn}}$ is a phenotype, ${\mathrm{Batch}}_{i}$ is the fixed effect of batch (by company), ${\mathrm{Age}}_{k}$ is the covariate effect of entry age, ${\mathrm{Pen}}_{l}$ is the random environmental effect of pen within batch, ${\mathrm{Litter}}_{m}$ is the random effect of litter, $u_{n}$ is the random additive genetic effect, and $e_{\mathrm{ijklmn}}$ is a residual.

Genomic relationship matrices for the within-population analyses were created using method I of VanRaden (2008). The pedigree included a total of 399,950, 533,565, 337,909, 378,103, and 163,114 pigs for LR1, LW1, LR2, LW2a, and LW2b, respectively. To estimate genetic correlations between a trait for a crossbred population (from Company 1 or 2) and one of its parental purebred populations, a multi-breed genomic relationship matrix was created following Wientjes et al. (2017):


$$[\begin{array}{cc} K_{11} & K_{12}\\ K_{21} & K_{22} \end{array}]=[\begin{array}{cc} \frac{M_{PB}M_{PB}^{'}}{\sum 2P_{j}^{PB}(1-P_{j}^{PB})} & \frac{M_{PB}M_{CB}^{'}}{\sqrt{\sum 2P_{j}^{PB}(1-P_{j}^{PB})}\sqrt{\sum 2P_{j}^{CB}(1-P_{j}^{PB})}}\\ \frac{M_{CB}M_{PB}^{'}}{\sqrt{\sum 2P_{j}^{PB}(1-P_{j}^{PB})}\sqrt{\sum 2P_{j}^{CB}(1-P_{j}^{PB})}} & \frac{M_{CB}M_{CB}^{'}}{\sum 2P_{j}^{CB}(1-P_{j}^{CB})} \end{array}],\;$$

where $K_{11}$ denotes relationships among the purebred animals, $K_{22}$ the relationships among the crossbred animals and $K_{21}$ and $K_{12}$ the relationships between purebred and crossbred animals, ***M***_*PB*_ (***M***_*CB*_) is a centered marker genotype matrix for the purebred (crossbred) population, and $P_{j}^{PB}$ ($P_{j}^{CB}$) is the allele frequency of marker *j* in the purebred (crossbred) population.


$$\mathrm{All}\;\mathrm{relationship}\;\mathrm{matrices}\;\mathrm{were}\;\mathrm{rescaled}\;\mathrm{using}:K^{'}=[\begin{array}{cc} \frac{K_{11}}{D_{K1}} & \frac{K_{12}}{\sqrt{D_{K1}}\sqrt{D_{K2}}}\\ \frac{K_{21}}{\sqrt{D_{K1}}\sqrt{D_{K2}}} & \frac{K_{22}}{D_{K2}} \end{array}],$$

w$\mathrm{ith}D_{K_{x}}=\overset{-}{\mathrm{Diag}(K_{x})}-\overset{-}{K_{x}}$, where $D_{K_{x}}$is a scaling factor for each matrix, $\overset{-}{\mathrm{Diag}(K_{x})}$ is the mean of diagonals in $K_{x}$, and $\overset{-}{K_{x}}$ is the mean of all elements in $K_{x}$.

Genetic parameters were estimated separately for each of the five purebred populations. Resulting estimates for the five purebred populations, and their standard errors, were then used in a random effects meta-analysis, following Borenstein et al. (2010), to derive an overall estimate for each parameter. In order not to overemphasize estimates at the boundaries, which can have small estimated standard errors, the minimum standard error assigned to individual estimates to derive weights for the meta-analyses was set equal to 0.05 for heritability estimates and 0.3 for genetic correlation estimates.

## Results

Table 1 shows summary statistics for the potential resilience indicators derived from feed intake of purebreds in high-health nucleus herds. Distributions are in Supplementary Figure S1. Means were consistent across populations for all three indicator traits (SD, AC, and SK), except for AC, which had an average of 0 in LW2b but was on average positive for the other four breeds (0.39 to 0.50). SK was on average slightly positive for four purebred populations (0.03 to 0.07), but slightly negative (−0.08) for LR2. Standard deviations of the resilience indicators were also consistent across breeds, except that the standard deviation of SK was greater for the two breeds from company 1 (0.56 for LR1 and 0.60 for LW1) than those for the three breeds from company 2 (ranging from 0.37 to 0.40).

Table 2 shows means and standard deviations for the traits recorded on crossbreds derived from these five purebred populations in the NDCM. These were similar to those presented by Cheng et al. (2020) for crossbred pigs from all seven breeding companies that provided pigs to the NDCM.

### Heritabilities

**Figure 1.**
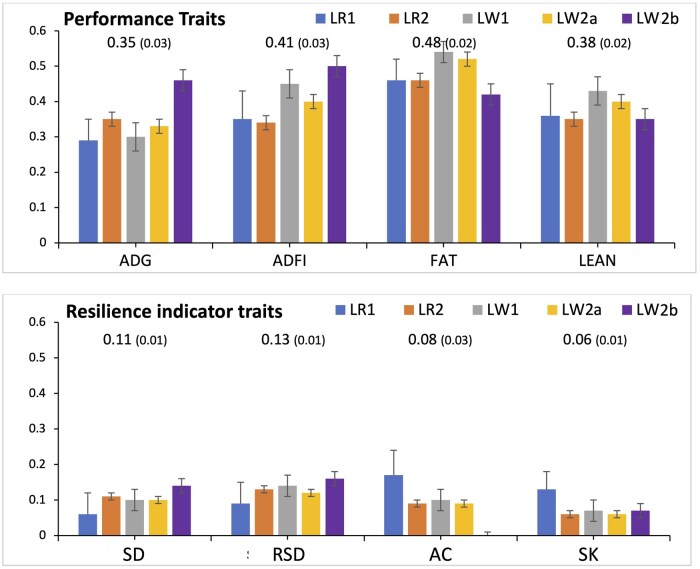
Estimates of heritabilities (and standard error bars) for the performance (average daily gain (ADG), average daily feed intake (ADFI), and ultrasound backfat (FAT) and lean depth (LEAN)) and resilience indicator traits (SD, RSD, AC, SK) for the five purebred populations (LR1, LR2, LW1, LW2a, and LW2b). Numbers in the graph are meta-analysis estimates, with their standard errors in brackets. Resilience indicator traits derived from residuals of linear regression of longitudinal feed intake on age: SD = standard deviation; RSD = square root of SD; AC = first order auto-correlation; SK = skewness.

Estimates of heritabilities for production and resilience indicator traits in the five purebred populations are shown in ­Figure 1. In general, heritability estimates for a given trait were similar for the five purebred populations. Heritability estimates were moderate to high for performance traits (0.22 to 0.54) and low to moderate for resilience indicator traits, with average estimates ranging from 0.06 for SK to 0.13 for RSD, which had a slightly higher heritability estimate than SD. The heritability estimate for AC for population LW2b, which had a much lower mean AC than the other four populations (Table 1), was essentially zero. Heritability estimates of performance and resilience (indicator) traits of crossbreds in the NDCM were provided by Cheng et al. (2020).

### Genetic correlations among resilience indicator traits in purebreds

**Figure 2.**
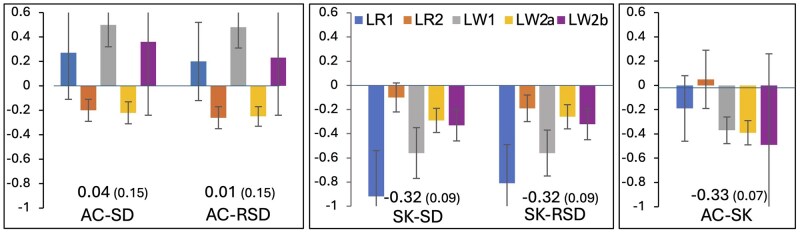
Estimates (and standard error bars) of genetic correlations between resilience indicator traits (AC, SK, and RSD) in the five purebred populations (LR1, LR2, LW1, LW2a, and LW2b). Correlations not shown for AC in LW2b because its heritability estimate was zero. Numbers in the graph are meta-analysis estimates, with their standard errors in brackets. Resilience indicator traits derived from residuals of linear regression of longitudinal feed intake on age: SD = standard deviation; RSD = square root of SD; AC = first order auto-correlation; SK = skewness.

Estimates of genetic correlations among AC, SK, and RSD in the five purebred populations are shown in Figure 2. SD had a very high genetic correlation estimates with RSD, with a weighted average of 0.99 (Supplementary Tables S1–S5), and these two traits also had very similar genetic correlation estimate with AC and SK. Therefore, and because RSD had higher heritability estimates than SD, only results for RSD will be shown in the remainder.

SK had negative or close to zero genetic correlation estimates with both RSD and AC for all purebred populations, averaging −0.32 and −0.33, respectively (Figure 2). Estimates of the genetic correlation between RSD and AC averaged 0 and were variable for the five purebred populations, positive for Company 1 (0.20 and 0.48) and for LW2b (0.23) of Company 2 but negative for LR2 and LW2a of Company 2 (both −0.26).

### Genetic correlations between resilience indicator traits and performance traits in purebreds

**Figure 3.**
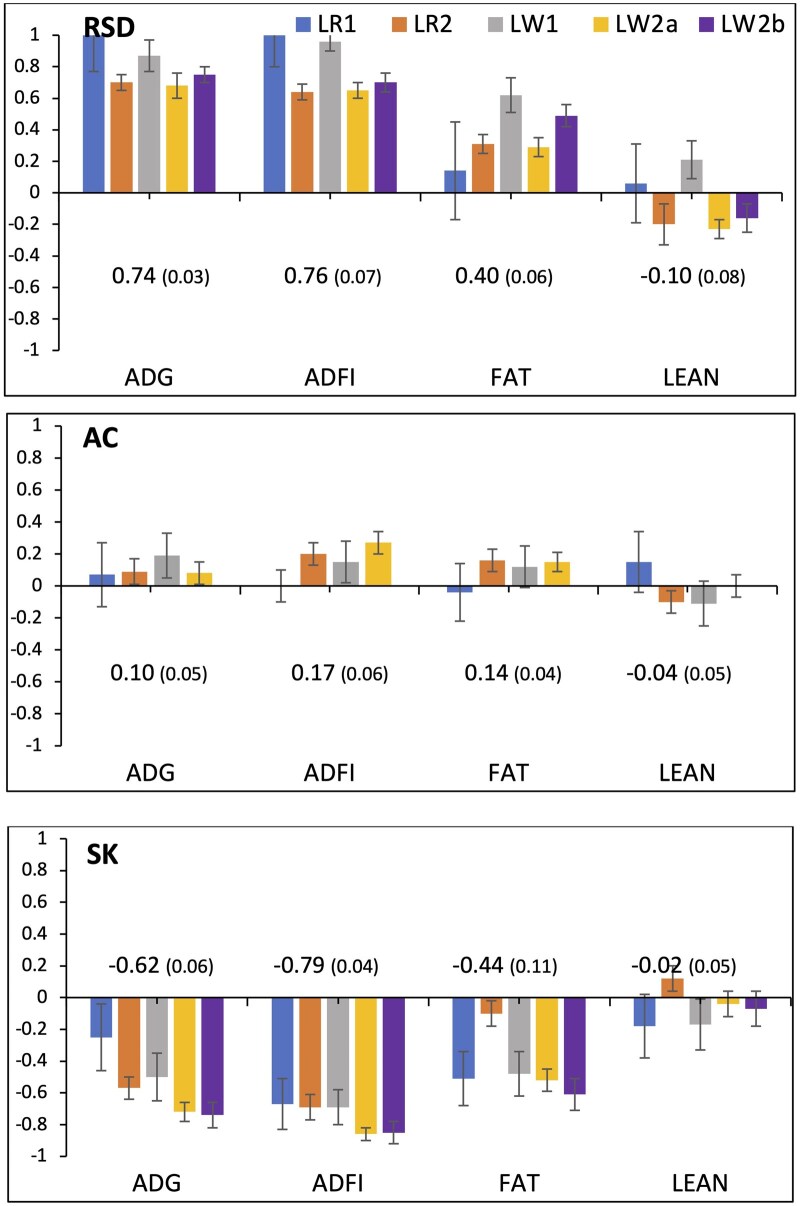
Estimates (and standard error bars) of genetic correlations of resilience indicator traits (AC, SK, and RSD) with performance traits in purebreds (average daily gain (ADG), average daily feed intake (ADFI), and ultrasound backfat (FAT) and lean depth (LEAN)) for the five purebred populations (LR1, LR2, LW1, LW2a, and LW2b). Correlations not shown for AC in LW2b because its heritability estimate was zero. Numbers in the graph are meta-analysis estimates, with their standard errors in brackets.

Estimates of genetic correlations between resilience indicator traits and performance traits in the five purebred populations are in Figure 3. SK exhibited consistent and substantial negative genetic correlation estimates for most purebred populations with ADG (−0.62 on average), ADFI (−0.79), and FAT (−0.44), while having close to zero estimates with LEAN. Corresponding genetic correlation estimates for RSD showed similar trends but in the opposite direction. RSD had notably high positive genetic correlation estimates with ADG and ADFI in the two purebred populations of Company 1 (1.00 for LR1 and 0.87 and 0.96 for LW1), while they ranged from 0.64 to 0.75 for the three purebred populations of Company 2. Estimates of genetic correlations between RSD and FAT were moderately positive for all purebred populations, averaging 0.40, but those between RSD and LEAN were again relatively close to zero. For AC, excluding estimates for LW2b, for which AC was determined to have a very low heritability, genetic correlation estimates were small positive with ADG (0.10 on average), ADFI (0.17), and FAT (−0.14), but fluctuated around zero for LEAN.

### Genetic correlations for performance traits between purebreds in the nucleus and crossbreds under a disease challenge

**Figure 4.**
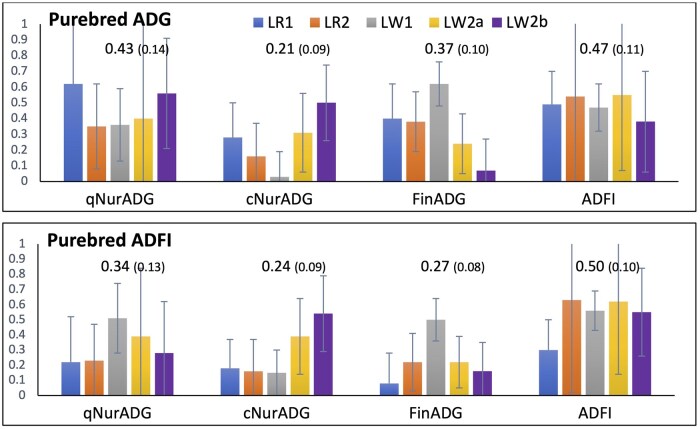
Estimates (and standard error bars) of genetic correlations for average daily gain (ADG) and average daily feed intake (ADFI) between nucleus purebreds and crossbreds in the quarantine nursery (qNur) and under a disease challenge in the challenge nursery (cNur) and finisher (FIN) for the five purebred populations (LR1, LR2, LW1, LW2a, and LW2b). Numbers in the graph are meta-analysis estimates, with their standard errors in brackets.

Estimates of genetic correlations for production traits between purebreds (ADG and ADFI) and crossbreds (qNurADG, cNurADG, FinADG, and ADFI) ranged from 0.03 to 0.63, as illustrated in Figure 4. Across the five purebred populations, estimates of genetic correlations of finisher ADG of purebreds with ADG of crossbreds in the quarantine nursery, challenge nursery, and finisher averaged 0.43, 0.21, and 0.37, respectively, and 0.47 with ADFI in the finisher. Corresponding average estimates for ADFI on purebreds were 0.34, 0.24, 0.27, and 0.50, respectively.

### Genetic correlations of resilience indicator traits between purebreds in the nucleus and crossbreds under a disease challenge

**Figure 5.**
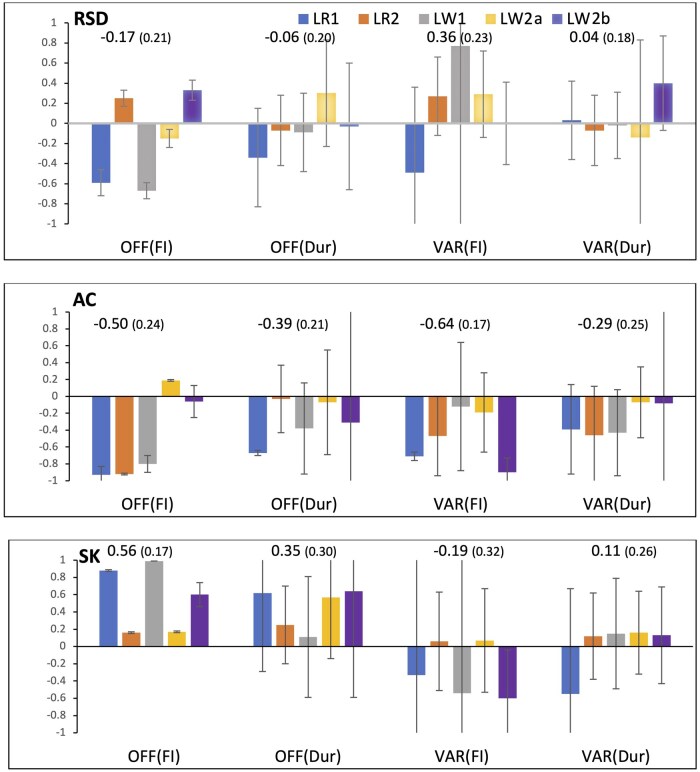
Estimates (and standard error bars) of genetic correlations of resilience indicators traits computed for purebreds (RSD, AC, SK) with disease indicator traits computed on crossbreds (OFF(FI), OFF(Dur), VAR(FI), and VAR(Dur)) for the five purebred populations (LR1, LR2, LW1, LW2a, and LW2b). Numbers in the graph are meta-analysis estimates, with their standard errors in brackets. Resilience indicator traits derived from residuals of linear regression of longitudinal feed intake on age: SD = standard deviation; RSD = square root of SD; AC = first order auto-correlation; SK = skewness.

Estimates of genetic correlations between resilience indicator traits derived from feed intake data on purebreds in the nucleus and the resilience indicator traits derived from feed intake data on crossbreds under a disease challenge proposed by Putz et al. (2019) are in Figure 5. Estimates of genetic correlations for RSD of purebreds with the proportion of off-feed days and SD of day-to-day variation in feed intake (equivalent to SD, as defined here for purebreds) on crossbreds under challenge were very variable, ranging from −0.67 to 0.77, and averaging −0.17 and 0.36, respectively. Corresponding genetic correlations of RSD with off-feed days and SD based on feeding duration were close to zero (averaging −0.06 and 0.04, respectively). Genetic correlation estimates of AC of purebreds with the four resilience indicator traits derived by Putz et al. (2019) on crossbreds tended to be moderately negative (averaging −0.29 to −0.64) but differed between purebred populations (close to zero for LW2a and LW2b for all crossbred traits, except for SD for LW2b (−0.90)). Corresponding estimates for SK on purebreds were moderately positive with off-feed days (on average 0.56 and 0.35 when based on intake and duration, respectively) and closer to zero or negative for SD based on intake (−0.19 on average) and duration (0.11 on average).

### Genetic correlations of resilience indicator traits in purebreds with performance of crossbreds under a disease challenge

**Figure 6.**
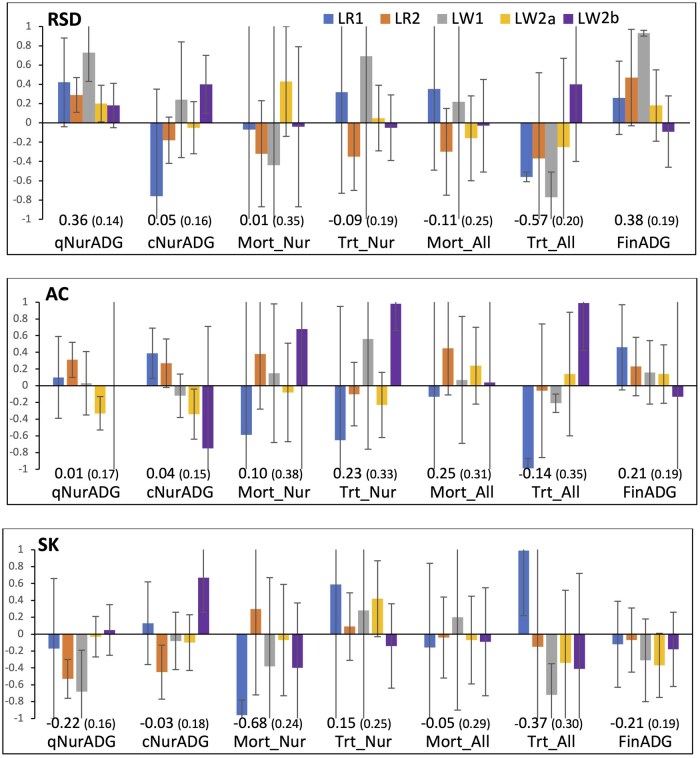
Estimates of genetic correlations of resilience indicators traits (RSD, ACF, SKEW) in purebreds with resilience traits (average daily gain (ADG), mortality (Mort), and veterinary treatment rate (Trt) of crossbreds in the quarantine nursery (qNur) and under a disease challenge in the challenge nursery (cNur), the finisher (FIN), and across the challenge nursery and finisher (All), and with disease indicator traits of crossbreds (OFF(FI), OFF(Dur), VAR(FI), and VAR(Dur)) for the five purebred populations (LR1, LR2, LW1, LW2a, and LW2b). Numbers in the graph are meta-analysis estimates, with their standard errors in brackets. Resilience indicator traits derived from residuals of linear regression of longitudinal feed intake on age: SD = standard deviation; RSD = square root of SD; AC = first order auto-correlation; SK = skewness.

Estimates of genetic correlations of resilience indicator traits of purebreds with performance and resilience traits of crossbreds under the challenge are shown in Figure 6. Estimates of genetic correlations of RSD on purebreds tended to be positive with ADG of crossbreds in the quarantine nursery (average 0.36) and in the finisher (0.38) but very variable and on average close to zero with ADG in challenge nursery (0.05 on average). Purebred resilience indicator trait RSD also tended to have negative genetic correlation estimates with veterinary treatments across the challenge (−0.57 on average) but its correlation estimates with mortality and with treatment rate in challenge nursery were highly variable and on average close to zero (0.01 and −0.09, respectively, on average).

Estimates of genetic correlations of AC in purebreds with performance of crossbreds under challenge were highly variable between the five purebred populations and were on average close to zero (Figure 6), except for LW2b. For this purebred population, estimates were high negative for ADG in the challenge nursery (−0.75 but with a very large SE of 1.46) and high positive for mortality in the challenge nursery (0.68 but again with a very large SE of 3.35) and for treatment rate in the challenge nursery and across the challenge (0.98 and 0.99 with SE of 0.32 and 0.57, respectively). Estimates were a bit more consistent across purebred populations for SK and they were on average negative with ADG in quarantine nursery (−0.22) and in the finisher (−0.21) but close to zero with ADG in challenge nursery (−0.03), negative with mortality in challenge nursery (−0.68) and with treatment rate across the challenge (−0.37), but slightly positive with treatment rate in the challenge nursery (0.15) and essentially zero with mortality across the challenge (−0.05).

## Discussion

Labor and health costs of pork production can be reduced and efficiency and animal welfare improved by breeding pigs that remain healthy and that are easy to manage. To this end, resilience, and specifically disease resilience, have been proposed as a desirable target for selection (Mulder and Rashidi, 2017; Knap and Doeschl-Wilson, 2020). Defined as the ability of an animal to be minimally affected by disease or to rapidly return to the state pertained before exposure to the disease (Albers et al., 1987; Colditz and Hine, 2016), measuring disease resilience is, however, difficult and is not possible in purebred nucleus breeding programs because of their requirements for high biosecurity. Scheffer et al. (2018) and Berghof et al. (2019b) suggested that longitudinal data recorded on individuals, such as body weight, milk yield, egg production, feed intake, water intake, and activity, can be used to derive measures that may be genetic indicators of resilience, specifically, measures that quantify some aspect of patterns or changes in the trait over time. For such measures to be effective genetic indicator traits to select for resilience, they must be heritable and genetically correlated with resilience. And, indeed, several studies have shown that such measures can be heritable (Gorssen et al., 2023) and related to resilience and, more specifically, with disease resilience, in dairy cattle (Poppe et al., 2020) and pigs (Putz et al. 2019; Cheng et al., 2020, Gorssen et al., 2024). van der Zande et al. (2020) used ear tag accelerometers to evaluate several dynamic measures of activity in nursery pigs before and after an experimental challenge with PRRS virus, of which some were found to be phenotypically correlated with clinical signs and mortality. As camera and sensor technology develops further for application in management and breeding (Pérez-Enciso and Steibel, 2021), many other longitudinal measures that may relate to resilience will become available and should be investigated.

Colditz and Hine (2016) broadened the definition of resilience to include responses to both infectious and non-infectious stressors because responses to these stressors share a common defense strategy. And indeed, in their review, Gimsa et al. (2018) documented the close connection between immune and stress response systems and the effect of psychosocial stress on innate and adaptive immune responses. Colditz and Hine (2016) also suggested to use standard husbandry practices that provide physical and social stressors to animals, such as weaning, as the basis of resilience phenotypes. Accordingly, Hermesch et al. (2017, 2018) showed that several immune and hematological traits measured on weaned pigs had moderate to high heritabilities and significant genetic correlations with subsequent growth traits.

Although nucleus breeding programs do not allow selection candidates to be exposed to disease, they are exposed to other stressors, including the stress of weaning, mixing, novel diets, human interaction, etc, and potentially heat stress. In addition, recording daily feed intake on individual boars during the test period (∼70 to 180 d of age) is now routine in most nucleus breeding programs. Thus, indicator traits derived from these longitudinal data may have potential as indicator traits to select for (disease) resilience under commercial circumstances.

Berghof et al. (2019b) proposed several measures derived from longitudinal data as potential resilience indicators. These were all based on daily deviations of phenotypes from expectation, derived as the residuals from a statistical model fitted to the longitudinal data of an animal. Proposed measures included day-to-day variation of deviations, skewness of deviations, and the lag-one auto-correlation of deviations over time (Berghof et al., 2019b). Berghof et al. (2019b) provided interpretations of these measures, which we applied under the assumption that all individuals within a batch are exposed to the same disturbances. Under this assumption, and following Berghof et al. (2019b), the standard deviation of deviations (SD) quantifies the impact of disturbances on the longitudinal phenotype, with animals that that are less affected by the disturbances, both in severity and duration, having a lower SD; the lag-one autocorrelation (AC) of deviations quantifies the duration of the impact of disturbances on the longitudinal phenotype, with pigs that are not affected by disturbances or that have a fast recovery from disturbances having an AC around 0, pigs that are affected by disturbances and have a slow recovery having a larger positive AC, and pigs that are affected by disturbances and have a fast and overreacting response having a larger negative AC; and skewness of deviations (SK), which quantifies the direction of the impact of the disturbances on the longitudinal phenotype, with pigs that have mainly negative deviations in the longitudinal phenotype in response to disturbances having negative SK, pigs that have mainly positive deviations having a positive SK, and pigs that are not affected by disturbances or that have both negative and positive deviations in response to disturbances having an SK around 0.

In our data, the average SD was very consistent, at 0.32 kg/day, for all five purebred populations, except for LW2a, which had an average SD of 0.35 (Table 1). The standard deviation of SD was also very consistent for all five populations (0.17 to 0.18 kg/day). The crossbred progeny of these lines had substantially greater SD (referred to as VAR_FI_ in Table 2) under the disease challenge, at 0.50 and 0.47 kg/day for Company 1 and 2, respectively, while the standard deviation of this measure was substantially smaller than in the purebred populations, at 0.10 and 0.11 kg/day for the two companies. While the higher average SD under the challenge may be a direct result of the impact of disease on feed intake, difference in feed intake recording and data editing procedures may also contribute to these differences. Using data from purebred Duroc pigs, Li et al. (2024) observed a substantially larger average SD (denoted as RMSE_FI_ in their publication) of 0.59 kg/day, with a slightly smaller standard deviation of 0.15 kg/day than observed in our purebred data. Kavlak and Uimari (2024), using reported a mean SD (RMSE_FI_) of 0.40 kg/day, in mostly purebred Yorkshire and Landrace pigs in a central test station, slightly larger than in our purebred data, with a standard deviation of 0.11 kg/day, which was substantially smaller than in our purebred data but similar to that observed in our crossbred data under challenge. The greater similarity of the mean and standard deviation of SD to our results under the disease challenge may correspond to the lower health status of the central test station, which represented a typical commercial setting in Finland (Kavlak and Uimari, 2024), compared to purebred nucleus herds.

Means for AC were substantially positive for all purebred populations, except for LW2b, which had an average AC of 0. Based on the interpretation of a positive AC by Berghof et al. (2019b), this indicates that pigs in four of the purebred populations were affected by disturbances and tended to have a slow recovery, compared to LW2b, which tended to be not affected by disturbances. Whether these differences are genetic or the result of environmental effects is not clear, although there were no notable differences in how pigs from LW2a and LW2b were housed and treated by company 2. Note, however, that AC had a very low heritability in LW2b (Figure 1), indicating substantial noise in the AC values for this population.

The mean for SK was slightly positive for four purebred populations (0.03 to 0.07), but slightly negative (−0.08) for LR2, suggesting that pigs from LR2 may, on average, be more affected by negative disturbances than pigs from the other four purebred populations. Again, it is not clear whether these population differences are genetic. Nevertheless, the limited skewness observed in these data also do not suggest that these pigs were substantially affected by disturbances, or that the disturbances were frequent or severe. It was also notable that the standard deviation of SK was greater for the two purebred populations from company 1 (0.56 for LR1 and 0.60 for LW1) than those for the three purebred populations from company 2 (ranging from 0.37 to 0.40). These differences may be the result of differences in feed intake recording and data editing procedures between the two companies. Comparative statistics for AC and SK are not available in the literature for pigs.

### Genetic parameters of resilience indicators among purebreds

In general, resilience indicators derived from longitudinal feed intake data on purebreds in nucleus herds were low to moderately heritable, averaging 0.13 for RSD, 0.08 for AC, and 0.06 for SK (Figure 1). Cheng et al. (2020) estimated the heritability of RSD to be lower (0.08) under a disease challenge, but substantially higher (0.23) when feeding duration rather than feed intake was used as the longitudinal phenotype. Unfortunately, feeding duration was not available for these purebred populations. These heritability estimates are at the low end of estimates obtained for measures of resilience based on feed intake in some other purebred nucleus populations (Homma et al., 2021; Gorssen et al., 2023) but similar to those obtained by Kavlak and Uimari (2024) and Mancin et al. (2024). Differences may be due to differences in recording and the specific feed intake resilience measurements that were analyzed. Although comparative statistics for AC and SK are not available in the literature for pigs, they have been explored in other species. Similar to our results, Poppe et al. (2020) also found low heritabilities for AC (∼0.09) and, in particular, for SK (<0.02) based on daily milk yield in dairy cattle. Estimates for body weight in layer chickens by Berghof et al. (2019a) were around 0.10 for all three resilience measures they examined, including ln(VAR), AC, and SK.

Genetic correlations among resilience indicators based on purebred nucleus data (Figure 2) tended to be close to zero between AC and RSD and negative (−0.32 on average) for SK with RSD and AC. This agrees with the conclusion of Berghof et al. (2019b) that these three measures potentially capture different aspects of resilience.

### Genetic correlations of production traits with resilience indicators in nucleus purebred data

To better understand the genetic relationship between production and resilience traits derived from feed intake in a high-health nucleus, genetic correlations between these respective sets of traits were estimated. RSD showed high positive genetic correlations with ADG and ADFI (Figure 3), especially in the two LR populations (>0.87) and ranging from 0.64 to 0.75 for the three LW populations. These genetic correlation estimates are substantially higher than the moderate genetic correlations that have been estimated for related pairs of traits in most previous studies for pigs (Gorssen et al., 2023; Mancin et al., 2024; Kavlak and Uimari, 2024), although Homma et al. (2021) also estimated genetic correlations for ln(VAR) for ADFI with ADG of up to 0.60 and up to 0.80 with ADFI. Poppe et al. (2020) also estimated large positive genetic correlations between ln(VAR) for milk production with milk production. It notable that, under a severe disease challenge, Putz et al. (2019) and Cheng et al. (2020) found SD to have a substantial positive genetic correlation with ADG in the challenge nursery, but low and even negative (but not significantly different from zero) genetic correlations for ADG in the finisher, noting that SD was derived based on ADFI in the finisher in these and the above literature studies. Thus, the genetic correlation of SD with ADG may depend on the severity and nature of the disturbances pigs that are exposed to.

Mancin et al. (2024) advocated for the use of resilience indicators that are more independent of other production traits and derived several novel measures based on feed intake data to achieve this. Although this may be desirable, genetic correlations among traits under selection can be accommodated by appropriate multi-trait genetic evaluation and selection approaches. Nevertheless, AC appears to be such a measure, as its genetic correlations with performance traits were essentially zero (Figure 3). In contrast, SK had substantial negative genetic correlations with ADG (−0.62 on average) and ADFI (−0.79 on average). The direction of these genetic correlations is as expected, as pigs with less positive skewness are expected to be less affected by disturbances (positive or negative) (Berghof et al., 2019b). Although the average SK was much lower for LW2b than for the other four populations, the genetic correlation of SK with production traits was not markedly different for that population.

### Genetic correlations of purebred nucleus performance with crossbred performance under a disease challenge

It is well known that traits recorded on purebreds in high-health nucleus herds can be genetically different than the same trait recorded on crossbreds in a commercial environment, as quantified by the genetic correlation between the same trait evaluated in these two conditions (Wientjes and Calus, 2017). While non-additive effects can contribute to this correlation being less than one, genotype by environment interactions can also contribute.

Our average estimates of the genetic correlation of ADG and ADFI measured on purebreds and the corresponding traits of ADG and ADFI in the finisher on crossbreds under the disease challenge of 0.37 and 0.50, respectively (Figure 4), are both at the lower end of estimates obtained in studies reviewed by Wientjes and Calus (2017). This is not surprising given the severe disease challenge that the crossbred pigs were exposed to, compared to the environment that crossbreds would be evaluated under in most literature studies. Although a meta-analysis of literature estimates by Wientjes and Calus (2017) suggested that non-additive genetic effects may be a more important reason than GxE why the genetic correlation between purebred and crossbred performance is less 1, it is clear that the extreme environment that the crossbreds were exposed to in this study will have been a major contributor.

### Genetic correlations of resilience indicator traits of purebreds with performance of crossbreds under a disease challenge

The ultimate goal of this study was to determine whether measures of resilience derived using feed intake data on purebreds in high-health nucleus herds could be used as genetic indicators to select for disease resilience of crossbreds in commercial herds. While the preliminary study by Harlizius et al. (2020) suggested that EBV of purebred boars for SD measures in nucleus herds are negatively correlated with disease resilience of their progeny, as expected based on the results of Putz et al. (2019) and Cheng et al. (2020) based on the genetic correlation of SD of feed intake with resilience within the disease challenge, our results were not able to confirm this relationship. In fact, although genetic correlations for RSD in purebreds with resilience traits of crossbreds under the challenge were highly variable and both positive and negative (Figure 6), the tendency was for higher RSD in purebreds to be associated with greater resilience under disease, based on the on average negative estimate of its genetic correlation with treatment rate across the challenge (−0.57) and its on average positive genetic correlation estimate with ADG in the finisher (0.38). The low genetic correlation between RSD on purebreds in the nucleus and RSD on crossbreds under challenge (ranging from −0.49 to 0.77 and averaging 0.37, Figure 5), likely is a strong contributor to these results. Thus, RSD on purebreds in the nucleus is genetically a substantially different trait than RSD on crossbreds under a severe disease challenge, with difference in disease pressure between these two environments likely being the major determinant of these differences. It should be noted, however, that Putz et al. (2019) and Cheng et al. (2020) found resilience traits derived based on feeding duration rather than intake under the challenge to be stronger genetic indicators of disease resilience. Unfortunately, feeding duration data were not available on purebreds in this study. Putz et al. (2019) and Cheng et al. (2020) also found resilience traits based on the proportion of off-days based on feed intake or feeding duration to be useful genetic indicators of resilience. These measures were not implemented on the purebred data because off-days were expected to be limited given the high-health status and high level of management in nucleus herds. However, RSD on purebreds also had very variable and on average close to zero estimates of genetic correlations with these resilience measures under challenge (Figure 5).

In addition to RSD, we explored the use of AC and SK as measures of resilience for purebred nucleus feed intake data, based on recommendations by Berghof et al. (2019b). Both these traits had more consistent genetic correlation estimates with the disease resilience indicator traits proposed by Putz et al. (2019) based on feed intake under challenge, that is, SD and off-days based on feed intake and duration (Figure 5). AC tended to have negative genetic correlations with each of these measures, suggesting that a higher AC in the nucleus is genetically associated with lower SD and off-days under challenge. Note that this is opposite to expectations based on Berghof et al. (2019b), who suggested that high AC is associated with greater susceptibility to disturbances and slower recovery. Estimates of genetic correlations of AC with disease resilience related traits under challenge were highly variable and on average close to zero (Figure 6) for the four purebred populations that had a positive average AC (Table 2) and for which the majority of animals had a positive AC (Supplementary Figure S1). However, for population LW2b, which had an average AC close to zero, genetic correlations of AC with disease resilience traits were in the expected direction (negative with ADG in the challenge nursery and positive with mortality and treatment rates) and substantial, although with large standard errors because AC was found to have a very low heritability in that population. So the relevance of these estimates is doubtful.

SK in purebreds tended to have positive genetic correlations with off-days of crossbreds under challenge (Figure 5), which is opposite to expectations, as a negative SK is expected to be associated with greater impacts of negative disturbances (Berghof et al., 2019b) but having more off-days reflects a greater impact of negative disturbances, that is, disease. However, higher SK in purebreds did tend to be genetically associated with lower mortality and treatment rates under the disease challenge, which is in the expected direction.

## Conclusions

This work presents the first comprehensive evaluation of traits derived from patterns in deviations of feed intake from expectations, using data collected on purebred pigs in nucleus herds, as potential genetic indicators of resilience of crossbreds under disease. This study was motivated by the importance of disease resilience as a target for genetic improvement, the inability to directly evaluate selection candidates for disease resilience because of the biosecure environment that they must be kept in, and the abundance of feed intake data that is collected routinely on selection candidates in these biosecure environments. Performance traits showed low genetic correlations between the purebred and crossbred data, indicating substantial GxE as a result of the large difference in disease pressure. Three measures derived from residuals of the linear regression of daily feed intake data on age were investigated, including the square root of the standard deviation of residuals (RSD), the lag-one autocorrelation of residuals (AC), and the skewness of residuals (SK). These three measures had fairly low estimates of heritability, especially SK, and had zero to moderate negative genetic correlation estimates with each other, indicating that they may capture different aspects of feed intake patterns. RSD was, however, highly positively genetically correlated with ADG and ADFI within the purebred populations. RSD had low genetic correlations with a similar measure computed from feed intake of crossbreds under challenge, which suggests that day-to-day variance of feed intake is a different genetic trait for purebreds in a nucleus environment compared to that of crossbreds under a severe disease challenge, likely mostly caused by the difference in disease pressure. Estimates of genetic correlations of the three resilience measures on purebreds with different resilience phenotypes of crossbreds under the disease challenge were highly variable and on average close to zero. We conclude that the potential purebred resilience indicator traits evaluated here should not be used to select for resilience to disease without additional research because of inconsistent results. However, under the right conditions, resilience measures derived from feed intake and behavior traits (e.g., based on duration) collected on purebreds in high-health nucleus herds can contain relevant information that is genetically correlated to disease resilience under commercial circumstances. Finding these measures requires further research.

## Supplementary Material

skaf357_Supplementary_Data

## Supplementary Data

*Conflict of interest statement*. Members of PigGen Canada, that is, the seven breeding companies, provided the pigs whose data were used in this study and geneticists of the companies were involved in the design of the natural disease challenge project. However, because of the design of the study, this did not lead to actual conflicts of interest. First, members of PigGen Canada were not involved in the management of pigs under the challenge, nor in the data analysis, or interpretation of results. In addition, because of the confounding between batch and company (pigs from a given batch originated from one company) and the dynamic nature of the disease challenge, by design, the study did not enable comparisons between companies to be made, which could lead to conflicts of interest. Instead, the entire focus was on analysis of differences between pigs within a batch, that is, on genetic differences among pigs within a company. None of the other co-authors had real or perceived conflicts of interest.

## Abbreviations:

AC: lag-one autocorrelation of residuals
ADFI: average daily feed intake
ADG: average daily gain
CDPQ: Centre de développement du porc du Québec
cNur: challenge nursery
EBV: estimated breeding value
FAT: ultrasound backfat
Fin: finisher
LEAN: ultrasound lean depth
LR: Landrace
LW: Large White
NDCM: natural disease challenge model
OFF_DUR_: off-feed proportion based on feeding duration
OFF_FI_: off-feed proportion based on feed intake
qNur: quarantine nursery
RSD: square root of SD
SD: root mean square error of residuals
SK: skewness of residuals
VAR_DUR_: day-to-day variation in feeding duration
VAR_FI_: day-to-day variation in feed intake
