# Supplementary material for: Daily feed intake patterns of purebred nucleus boars as genetic indicators for disease resilience of crossbred barrows under a natural polymicrobial disease challenge
Source: J Anim Sci. 2025 Oct 15;104:skaf357. doi: 10.1093/jas/skaf357 (PMC12923160; doi:10.1093/jas/skaf357)
Supplement: skaf357_Supplementary_Data [file skaf357_supplementary_data.zip › Supplm Fig 1.pptx]

## Slide 1
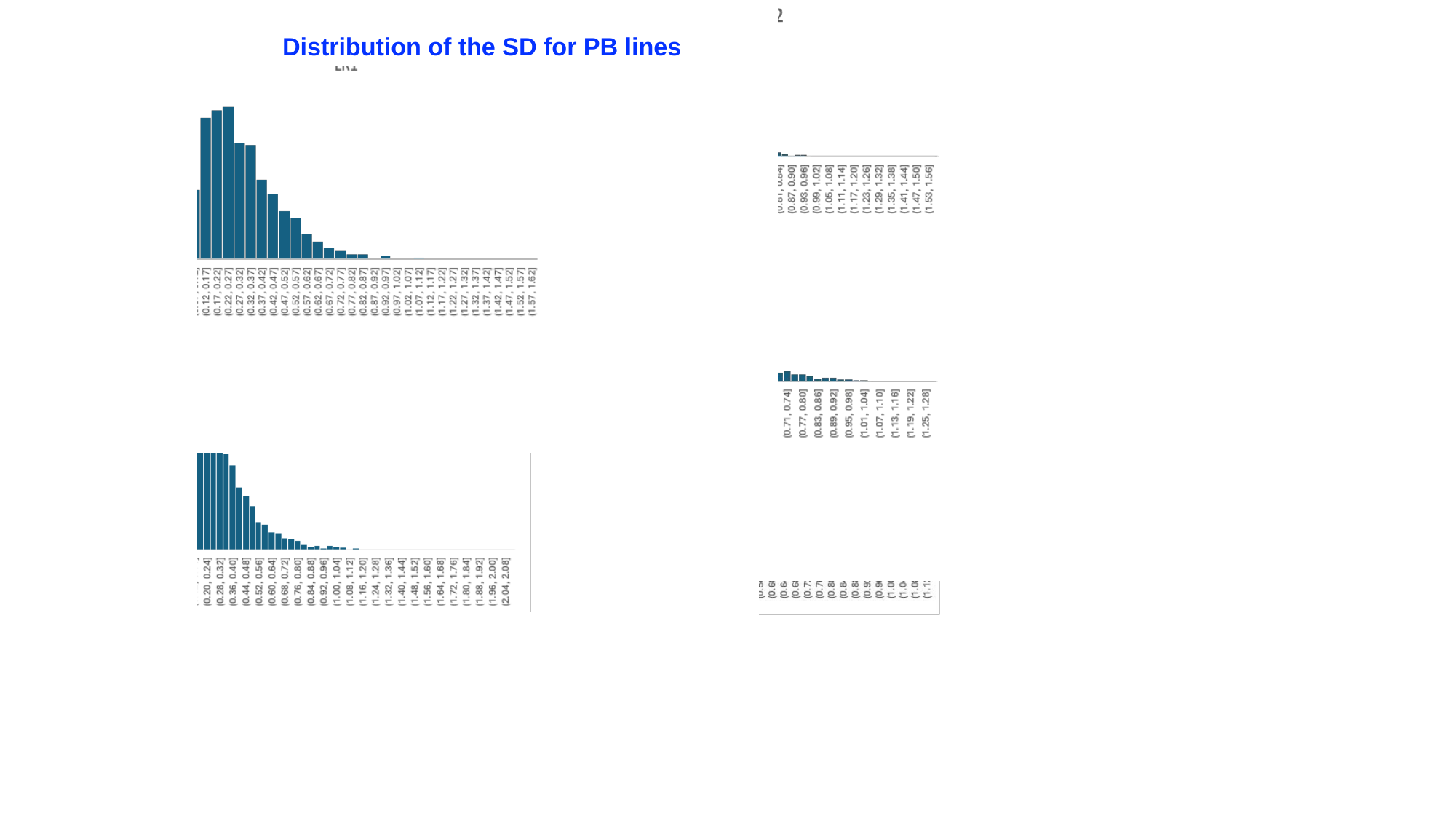

Distribution of the SD for PB lines

## Slide 2
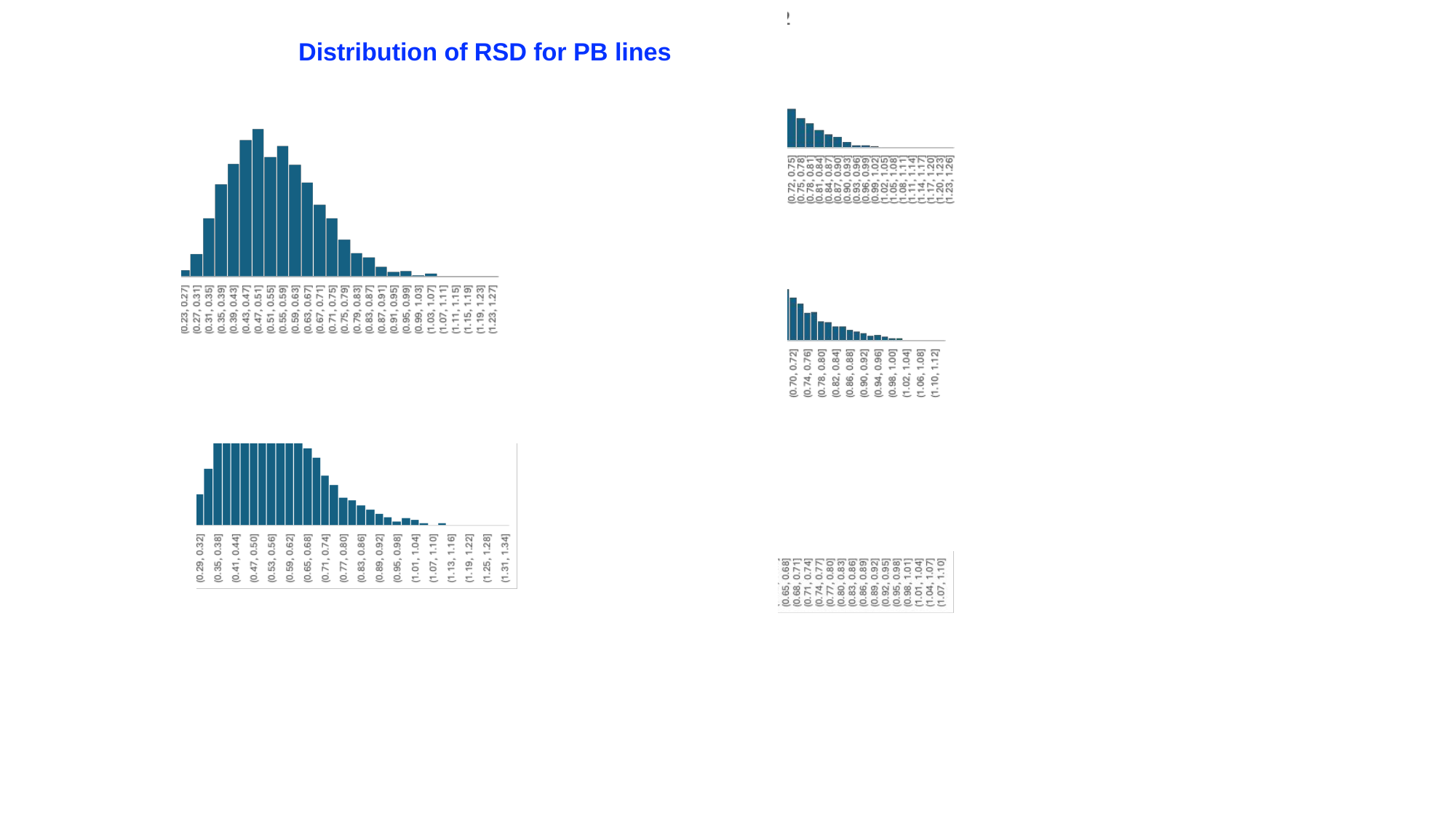

Distribution of RSD for PB lines

## Slide 3
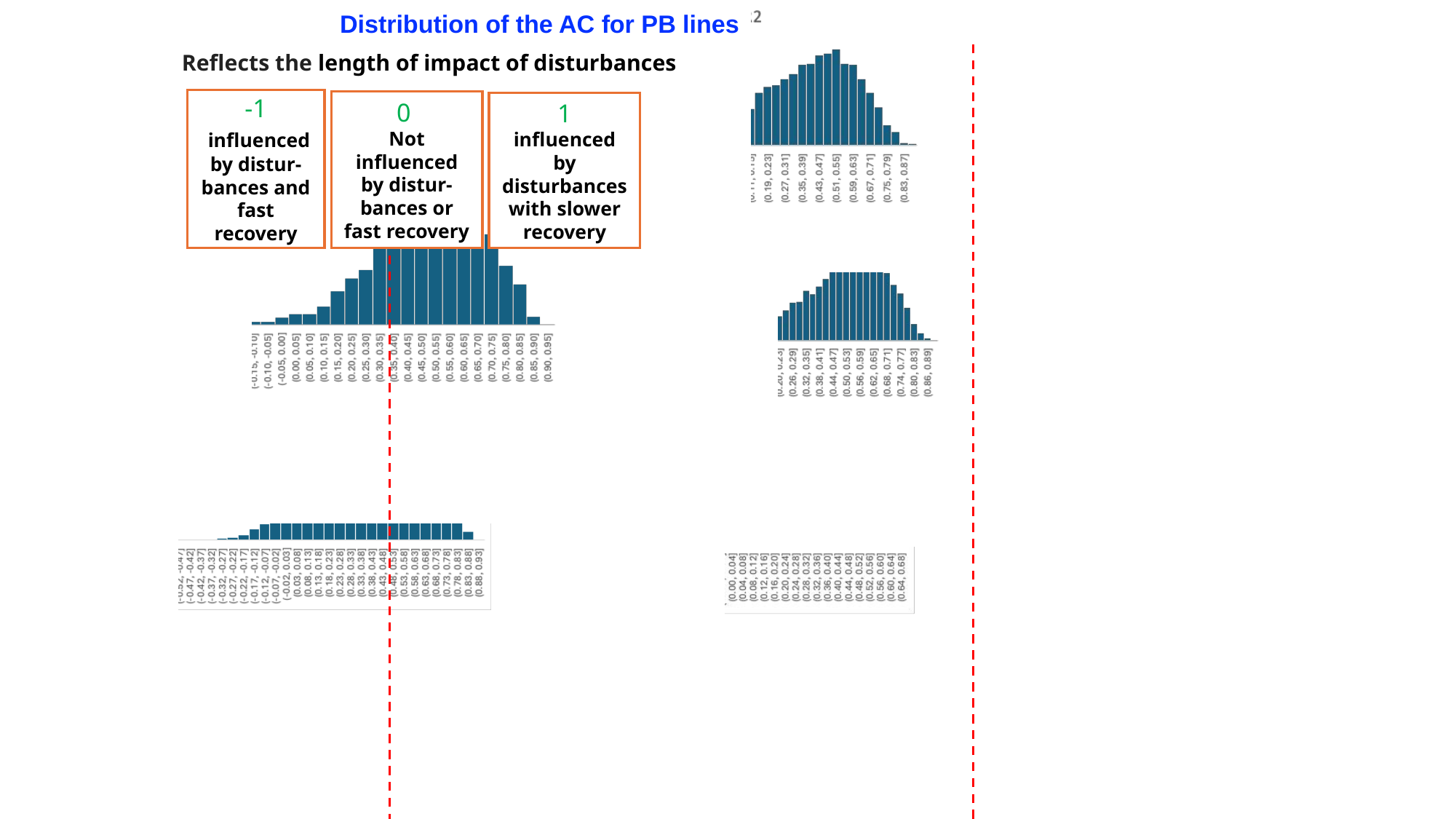

Distribution of the AC for PB lines
Reflects the length of impact of disturbances
-1
 influenced by distur-bances and fast recovery
0
Not influenced by distur-bances or fast recovery
1
influenced by disturbances with slower recovery

## Slide 4
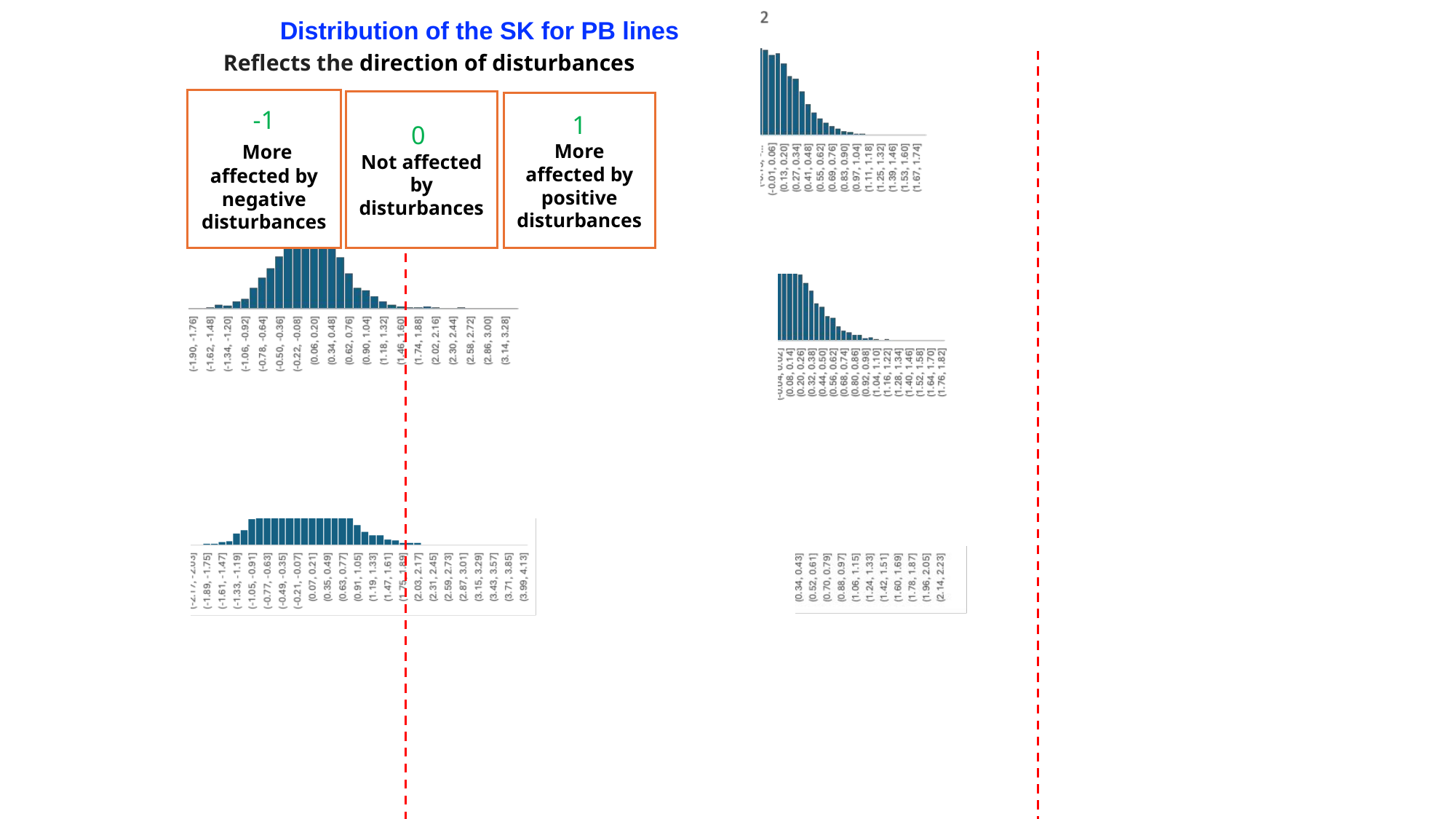

Distribution of the SK for PB lines
Reflects the direction of disturbances
-1
 More affected by negative disturbances
0
Not affected by
disturbances
1
More affected by positive disturbances
